# Factors affecting the transition to adulthood of Korean young adults with spina bifida: a qualitative study

**DOI:** 10.1186/s12912-023-01194-z

**Published:** 2023-02-20

**Authors:** Eun Kyoung Choi, Jisu Park, Kyua Kim, Eunjeong Bae, Yoonhye Ji, Seung Hyeon Yang, Altantuya Chinbayar, Hyeseon Yun

**Affiliations:** 1 College of Nursing, Mo-Im Kim Nursing Research Institute, Yonsei University, Seoul, Republic of Korea; 2 Department of Nursing, Yonsei University Graduate School, Seoul, Republic of Korea; 3 College of Nursing and Brain Korea 21 FOUR Project, Yonsei University, 50-1, Yonsei-ro, Seodaemun-gu, 03722 Seoul, Republic of Korea

**Keywords:** Focus groups, Qualitative research, Spina bifida, Transition, Young adult

## Abstract

**Background:**

Transition to adulthood to live independently while self-managing health and daily life without parental help is crucial for young adults with chronic conditions. Despite its importance as a precursor to effectively managing lifelong conditions, little is known about the experiences of young adults with spina bifida (SB) in transition to adulthood in Asian countries. This study aimed to explore the experiences of Korean young adults with SB to identify the facilitators or barriers to the transition from adolescence to adulthood from their perspectives.

**Methods:**

This study used a qualitative descriptive design. The data were collected in South Korea through three focus group interviews with 16 young adults with SB, aged 19–26, from August to November 2020. We conducted a qualitative content analysis using a conventional approach to identify the factors that facilitated and hindered the participants’ transition to adulthood.

**Results:**

Two themes emerged as facilitators and barriers to the transition to adulthood. a) Facilitators: understanding and acceptance of SB, acquiring self-management skills, parenting styles encouraging autonomy, parents’ emotional support, school teachers’ thoughtful consideration, and participation in self-help groups. b) Barriers: overprotective parenting style, experience of being bullied by peers, damaged self-concept, concealing one’s chronic condition from others, and the lack of privacy in school restrooms.

**Conclusions:**

Korean young adults with SB shared their experiences of struggling to properly manage their chronic conditions on their own, particularly concerning regular bladder emptying, during the transition from adolescence to adulthood. To facilitate the transition to adulthood, education on the SB and self-management for adolescents with SB and on parenting styles for their parents are important. To eliminate barriers to the transition to adulthood, improving negative perceptions of disability among students and teachers and making school restrooms CIC-friendly are needed.

**Supplementary Information:**

The online version contains supplementary material available at 10.1186/s12912-023-01194-z.

## Background

Spina bifida (SB) is a congenital neural tube defect caused by the incomplete closure of one or more vertebrae early in fetal development [1]. Depending on the level of damage to the spinal cord, SB can cause permanent dysfunction, such as neurogenic bladder or bowel, gait disturbance, and variations in cognitive processing [1, 2]. SB may require highly complex daily self-management, such as clean intermittent catheterization approximately 4–6 times a day, regular enema, and daily monitoring of skin integrity to prevent pressure ulcers owing to dull or no sensation in the lower extremities while wearing orthosis or using a wheelchair [1]. Although SB is a congenital malformation, most children with SB can survive and grow into adulthood; therefore, SB is considered a chronic condition that requires lifelong health management [3].

Recent studies on the long-term outcomes of adults with SB have reported increased bladder or bowel incontinence, obesity, hypertension, and chronic pain, resulting in secondary complications such as cardiovascular disease, pressure ulcers, and decreased health-related quality of life [4, 5]. However, most of these secondary conditions can be prevented; if not, at least their progression can be delayed with good self-care [5]. This suggests that it is important to properly manage one’s own medical condition continuously from childhood to adulthood [5]. For this, the transition to adulthood is important. Herein “transition to adulthood” means transfer of the primary responsibility for daily health self-management from parents to child and involves performing self-management and daily activities independently. It is critical for maintaining optimal lifelong well-being, particularly for those with childhood-onset chronic conditions [6]. Above all, the transition to adulthood should be planned in advance and prepared step by step [6, 7]

In particular, adolescence is a crucial developmental stage during the transition to adulthood because of the increased independence and autonomy and the gradual transfer of self-management responsibilities from their parents [7, 8]. Adolescence is the time to focus on forming one’s self-concept, expanding social relationships, and preparing for the future [9, 10]. Previous studies have pointed out that adolescence is when transition begins and requires intervention [6, 10–12]. Thus, in Western countries, many transition support programs and clinical practices are currently available for adolescents with SB [10–13]. On the contrary, in Korea, there are few transition support programs for adolescents and young adults with SB [14]. However, the findings from the previous study regarding needs of parents who have adolescents with SB in Korea suggest developing supportive programs to enable adolescents with SB to prepare for the future regardless of the disability [15].

To date, the factors influencing the transition to adulthood among adolescents with SB are known to vary by individual, developmental, clinical, psychosocial, parental, environmental, and cultural characteristics, including self-management, motivation, responsibility, independence, parenting styles, parents’ insight or role, condition-specific characteristics, and interpersonal relationship [6–12]. In particular, interventions to support the transition from adolescence to adulthood are known to be influenced by multifaceted factors according to the socio-ecological framework [12]. Given the influence of socio-ecological factors on the transition process [12], research is needed to explore these transition experiences and identify the influencing factors within the Korean sociocultural background.

Korea belongs to the east Asian culture that is collectivist, viewing self-concept from an interdependent perspective [16]. In the collectivist culture, people with disabilities report more difficulties due to the social stigma toward disabilities [16]. Additionally, adolescents with SB have described experiencing a constant struggle between concealing and disclosing their health conditions to others [17–19]. Considering the cultural impact on the transition to adulthood [10], it is unknown how collectivist cultural characteristics, such as social stigma related to disabilities or having a different appearance, might have negative influence on the transition of adolescents with SB. Therefore, this study explored the experiences of Korean young adults with SB, aiming to identify facilitators of or barriers to the transition from adolescence to adulthood from their perspectives.

## Methods

### Study design

This study employed a qualitative descriptive design using focus group interviews (FGIs) to address the research questions [20, 21]. This qualitative approach was selected as a means of obtaining a direct description of the specific phenomenon from participants’ perspectives [20]. This study adhered to consolidated criteria for reporting qualitative research (COREQ) checklist [22] (Additional file 1).

### Study setting

This study was performed in the Severance Children’s Hospital, which has the largest outpatient SB clinic of a tertiary hospital in Korea. The SB clinic has a multidisciplinary care system that consists of pediatric neurosurgery, orthopedic surgery, general surgery, and urology and provides diagnosis, examination, treatment, surgery, and follow-up for people with SB.

### Participants

The eligibility criteria for participants were: being 19–29 years of age, having a diagnosis of SB, and being capable of verbal communication. We confirmed the diagnosis of participants through electronic medical records. Individuals with cognitive impairment or other congenital malformations such as congenital megacolon were excluded.

### Sampling and recruitment

Purposive sampling was used until data saturation, and the participants were recruited through two channels: identification through a patient dataset as well as a pediatric nurse practitioner from an outpatient SB clinic. First, participants who were included in the patient dataset from the first author’s earlier study [23] and consented to being contacted for a follow-up study received an invitation by text message. Second, a pediatric nurse practitioner in the participating SB clinic introduced the study to patients regularly visiting the clinic. After being referred through either method, interested individuals contacted the first author. Finally, the first author contacted those who wanted to participate via telephone to explain the study’s aims and methods. The first author also explained to participants that there was no penalty for not participating in the study and that the medical staff would not know who participated in the study.

### Data collection

Data collection was conducted from August to November 2020 using semi-structured face-to-face FGIs. FGIs are particularly suitable when participants have a common background, as group interactions can help participants clarify their experiences [21]. An interview guide that focused on identifying the factors that facilitate or hinder the transition to adulthood of the participants was developed. The questions were as follows: 1) Would you kindly tell us about your transitional experiences from adolescence to adulthood?; 2) What were some of the difficulties you experienced during the transition to adulthood? If you encountered difficulties during transition to adulthood, could you tell us how you dealt with them?; 3) Were there any events or circumstances that positively affected your transition to adulthood? Were there any events or circumstances that negatively affected your transition to adulthood?; and 4) What kind of help do you need most for a transition to adulthood independently performing daily self-management? The FGI facilitator used probing questions to elicit further descriptions and details about the participants’ experiences, such as “Would you elaborate?” or “Would you explain using an example?” During the interviews, the meaning of “transition to adulthood” was explained as performing self-management and daily activities without parental help.

Three FGIs were conducted at a private seminar room in the College of Nursing of Yonsei University, which was a location familiar and accessible to all participants, as it is near the SB clinic. Only researchers and participants were involved in the FGIs, and each interview lasted for 90 to 120 min. The principal investigator served as the facilitator for all FGIs. Focus groups were divided by gender and consisted of five to six participants per group to facilitate interactions that could capture their diverse experiences [21]. The first group consisted of six young men, and the second and the third groups each comprised five young women. First, we interviewed a group of men, then a group of women, and similar factors influencing their transition to adulthood were derived by overlapping between the two interviews. However, the research team discussed that the second group of women had weak group interactions and the participants tended to respond passively; thus, the third group was interviewed. In the third active FGI, the research team discussed and confirmed the repetition of the contents of the two previous FGIs. At the time of the study, we considered data saturation to have been reached. During the FGIs, other researchers captured the group interactions through field notes that identified non-verbal cues and episodes of consensus and discord. All FGIs were audio-recorded using mini recorders and transcribed verbatim, while maintaining anonymity. Personal information was encoded in the transcription and analysis to prevent participant identification. All participants were provided a compensation of 50,000 KRW (about USD $40) after the FGIs.

### Data Analysis

Data were analyzed using qualitative content analysis through a conventional approach [24, 25]. In this analytic approach, we focused on identifying factors that facilitated or hindered the transition to adulthood. Two researchers (H.Y. and J.P.) independently analyzed transcripts, conducted open coding, and resolved any disagreements via discussion. Both repeatedly read all three transcripts for accuracy and immersed themselves in the data by reading transcripts several times to understand the overall meaning of the data. The key phrases in the transcripts were highlighted and summarized to form codes that were consistent with the participants’ statements. Finally, a bilingual researcher (K.K.) translated the participants’ quotations to retain vivid expressions and convey the meaning of the original language. We abstracted similar codes to form subcategories; subsequently, subcategories were grouped into categories. Some categories with common attributes were abstracted to form subthemes. Subthemes were then abstracted to themes and discussed with the research team to reach a consensus [24]. The identified themes adhered to the language of the participants as much as possible and conveyed an underlying pattern discerned from the data [26]. The first author (E.K.C.), an expert in clinical practice and research on SB, contributed to the final analysis and validation of the findings. The entire research team agreed on the final findings.

### Rigor

The rigor for the qualitative research was assured by the following Lincoln and Guba’s criteria of credibility, transferability, dependability, and confirmability [27]. To achieve credibility, i.e. internal validity [27] more than one data coder involved in the process of data analysis. For transferability, that is, whether or to what extent the findings are applicable within other contexts, circumstances, and settings [27], thick descriptions were provided. To enhance dependability, that is, reliability, which demonstrates the consistency and reliability of the study [27], audio-recorded and field notes were made during the FGIs to capture the participants’ verbal and non-verbal data. In terms of confirmability, which pertains to the fact that the qualitative research is not influenced by the researchers’ assumptions or biases [27], we used the actual participant quotes to describe the themes, and we audited whether the findings and interpretations were supported by data. The first author had previously conducted multiple qualitative and mixed-method studies, and she worked at the SB clinic as a pediatric nurse practitioner for eight years.

### Ethical considerations

Ethical approval was obtained from the Yonsei University Health System, Severance Hospital, Institutional Review Board (No. 4–2020-0624). All participants were informed about the purpose and method of this study, and written informed consent was obtained. They were assured of anonymity, confidentiality, and voluntary engagement and their ability to withdraw from this study at any time if they wished. We conducted the study in accordance with the Declaration of Helsinki.

## Results

A total of 16 young adults with SB participated in the study. The participant characteristics are presented in Table 1. The participants’ age was ranging between 19 and 26 years, and six men (37.5%) were included. Only one participant (6.3%) had a job, and the others were college students or jobseekers. Six (37.5%) were diagnosed with myelomeningocele, and 10 (62.5%) were diagnosed with lipomyelomeningocele. Only two (12.5%) had a ventriculoperitoneal shunt. Only one participant (6.3%) was a community ambulator (i.e., walking with or without crutches or braces and using a wheelchair only for long distances [28]), while the others were normal ambulators (i.e., walking without crutches or braces [29]). Regarding the method of bladder or bowel emptying, 13 (81.3%) used clean intermittent catheterization (CIC) and five (31.5%) used an enema. Regarding incontinence, seven (43.8%) experienced urinary incontinence, and six (37.5%) experienced fecal incontinence.

**Table 1:** Participant characteristics (*n* = 16)

Pseudonym	Gender	Age (years)	Career	Type of SB	VP Shunt	Method of Bladder Emptying	Method of Bowel Emptying	Incontinence		Ambulatory status
								Urinary	Fecal	
Alex	Man	21	Student	LMMC	No	CIC	Spontaneous	Yes	No	Normal ambulator
Benjamin	Man	19	Job- seeker	LMMC	Unknown	CIC	Spontaneous	Yes	Yes	Normal ambulator
Charles	Man	25	Student	LMMC	No	CIC	Spontaneous	No	No	Normal ambulator
Daniel	Man	23	Student	MMC	Unknown	Spontaneous	Spontaneous	Yes	No	Normal ambulator
Ethan	Man	20	Having a job	MMC	No	CIC	Enema	No	No	Normal ambulator
Fredrick	Man	23	Student	MMC	Yes	CIC	Spontaneous	Yes	Yes	Normal ambulator
Gloria	Woman	19	Student	LMMC	No	CIC	Enema	No	Yes	Normal ambulator
Helen	Woman	22	Student	LMMC	No	CIC	Enema	No	No	Normal ambulator
Irene	Woman	23	Student	LMMC	No	CIC	Spontaneous	No	No	Normal ambulator
Jane	Woman	20	Student	LMMC	No	CIC	Spontaneous	No	No	Normal ambulator
Kara	Woman	22	Student	MMC	No	CIC	Spontaneous	Yes	Yes	Normal ambulator
Luna	Woman	24	Student	LMMC	No	CIC	Spontaneous	No	No	Normal ambulator
Mia	Woman	21	Student	LMMC	No	Spontaneous	Spontaneous	No	No	Normal ambulator
Nora	Woman	26	Job- seeker	MMC	No	CIC	Enema	Yes	Yes	Normal ambulator
Olga	Woman	25	Job- seeker	MMC	Yes	Spontaneous	Spontaneous	No	Yes	Normal ambulator
Pamela	Woman	21	Student	LMMC	No	CIC	Stoma	Yes	No	Community ambulator

*CIC* Clean intermittent catheterization, *LMMC* Lipomyelomeningocele, *MMC* Myelomeningocele, *SB* Spina bifida, *VP shunt* Ventriculoperitoneal shunt

Two main themes emerged from qualitative content analysis: a) facilitators of the transition to adulthood and b) barriers to the transition to adulthood. Each theme had several subthemes, which included several categories (Table 2). Each category was described using quotes including the participants’ pseudonyms and age.

**Table 2:** Overview of themes and subthemes

Themes	Subthemes	Categories
Facilitators of the transition to adulthood	Individual factors	• Understanding and acceptance of SB • Acquiring self-management skills, particularly for bladder emptying
	Parental factors	• Parenting styles encouraging autonomy • Parents’ emotional support
	Interpersonal factors	• School teachers’ thoughtful consideration • Participation in self-help groups
Barriers to the transition to adulthood	Parental factors	• Overprotective parenting style
	Interpersonal factors	• Experience of being bullied by peers
	Sociocultural factors	• Damaged self-concept • Concealing one’s chronic condition from others • Lack of privacy in school restrooms

### Theme 1: facilitators of the transition to adulthood

This theme included factors that may facilitate the transition to adulthood from the perspective of young adults with SB in Korea.

#### Individual factors

##### Understanding and acceptance of SB

Participants discussed that accurately understanding SB and accepting it as a part of life was the starting point for enabling the transition to adulthood. Being aware that SB is a chronic condition made them realize that it is important to manage their own health in order to maintain optimal health conditions as much as possible throughout life.

*To transition, I need to fully prepare myself with condition-related knowledge and put effort into knowing myself. (Kara, 22)*

*I think the first thing is to realize that we have to live with this condition. (Nora, 26)*

##### Acquiring self-management skills, particularly for bladder emptying

All participants emphasized that acquiring self-management skills, particularly for CIC, as early as possible and making a habit of these skills were pivotal for their transition. Learning how to perform CIC at a young age helped them cope with problems caused by bladder dysfunction in daily life and interpersonal relationships, facilitating the transition.

*I learned CIC when I was seven years old. I figured out my own way of performing CIC. After I adjusted to living this way, there was not much trouble. (Luna, 24)*

*I think the younger you are, the better. The most important thing is to make CIC a habit. (Daniel, 23)*

#### Parental factors

##### Parenting styles encouraging autonomy

Parents who encouraged their children to be independent with regard the development of self-management skills, scheduling outpatient visits, and going to the hospital provided a foundation for participants’ independent growth. When parents raised their children in a way that emphasized autonomy, the transition was facilitated by self-management starting at a younger age.

*My mother raised me strong. She always said, “if you want something, you should do it by yourself.” I have been going to the hospital alone since I was in high school. (Irene, 23)*

##### Parents’ emotional support

Parents encouraged their children to do whatever they wanted despite their SB and motivated them to develop important values and beliefs. Parents’ emotional support made them confident, and confidence was the basis for self-management. This facilitated the transition to adulthood.

*My parents always told me, “Just need to do what you want to do. Your condition cannot hold you back from anything” (Daniel, 23)*

*My mom always reminded me whenever I started the semester, “You are not different from other students at all. What others can do, so can you.” (Irene, 23)*

#### Interpersonal factors

##### School teachers’ thoughtful consideration

Thoughtful consideration included class teachers informing other teachers about the participants’ condition and seeking their cooperation. This made it easier for the participants to self-manage in school and eventually became a factor in facilitating the transition.

*The most important thing is to meet a good class teacher. If I have a good class teacher, then everything goes well at school. (Benjamin, 19)*

##### Participation in self-help groups

A self-help group was formed by participating in an SB camp hosted by the hospital for children and adolescents with SB. Through the self-help group, the participants realized that they were not the only ones experiencing difficulties with SB. In addition, they received empathy and courage from seniors who had achieved successful transition beyond SB. It was also a great opportunity to learn about the condition and self-management. This positive self-reflection facilitated the participants’ transition.

*I thought I was living in this world alone. However, SB camp turned my whole world around and made me realize that I was not alone. I got to know my disease better at that camp. (Nora, 26)*

*The camp I went to when I was 17 was the turning point in my life. I met friends of my age and, for the first time, I realized that there were many others like me in this world. I changed my mind at that point, and I became more positive about my own life. (Pamela, 21)*

### Theme 2: Barriers to the transition to adulthood

This theme included factors that may hinder the transition to adulthood from the perspective of young adults with SB in Korea.

#### Parental factors

##### Overprotective parenting style

Overprotective parents still treated the participants as children with regard to caregiving, even though they were old enough to perform self-management. It made the participants dependent on their parents in performing self-management until adulthood, which hampered the transition to adulthood.

*My mother still treats me like a baby. She goes to the hospital with me and takes care of everything for me. I’ve heard many comments from others that I am too naïve. (Helen, 22)*

#### Interpersonal factors

##### Experience of being bullied by peers

Many participants experienced bullying and teasing when they were elementary- and middle-school students as they were different from others, for example, because they had to wear braces and perform CIC in order to urinate. This experience hurt them emotionally and intimidated them psychosocially. They struggled to conceal their disabilities from others, and even chose not to perform CIC at school at all. Thus, the transition to adulthood was hindered.

*After they knew about my condition, they spread rumors and bullied me. After that, I did not perform CIC when I was with my friends. As a result, I had to undergo kidney surgery once. (Benjamin, 19)*

*I couldn’t overcome my bullying experience. It was such a hard time for me. (Daniel, 23)*

#### Sociocultural factors

##### Damaged self-concept

Thoughts about being different from their friends without disabilities made participants miserable during adolescence. Some participants shared their experiences of despair when urine or feces leaked out unexpectedly while trying not to urinate at school. In addition, girls were especially sensitive about the shape of their legs and feet, walking posture, and surgical scars compared to boys. When they worked up the courage to wear a skirt, they had to endure curious gazes, which hurt their body image. These experiences ultimately hindered the transition to adulthood by lowering their self-concept, including self-esteem, self-efficacy, and body image.

*Whenever I made a mistake (incontinence), I thought that “Ah…other friends just control it (urination) naturally, why am I like this?” I became timid… (Olga, 25)*

*When I wear a skirt and braces and go outside, everyone stares at my legs shamefully. (Pamela, 19)*

##### Concealing one’s chronic condition from others

Some participants tried to hide details about their condition and CIC because they feared that their friends would bully them or spread rumors. Moreover, they had trouble self-managing at school while concealing their condition. This is an example of difficulties in daily life and social relationships acting as a factor in delaying the transition to adulthood.

*During youth, since even a little thing different from others led to teasing, I never told anyone that I did CIC. I had to make excuses every time I did CIC. Whether meeting friends or going on a trip, I thought a lot about when and where to go to the toilet. (Alex, 21)*

##### Lack of privacy in school restrooms

Participants desperately needed privacy-secured restrooms to perform CIC at school. However, many schools did not have toilets for people with disabilities. Boys, especially, had difficulties performing CIC at school because there were no separators between the toilets. For this reason, some participants failed to perform CIC or avoided performing CIC at school, which delayed the transition to adulthood.

*Especially when I was in middle school, other boys would kick the restroom door and would climb over the wall to see who was in the toilet. (Daniel, 23)*

*There were no disabled toilets in our school. I didn't even drink water to avoid CIC at school. (Nora, 26)*

## Discussion

Through FGIs, our findings identified the influencing factors that facilitated or hindered transition into independent living among young adults with SB. Most participants in this study had a mild type of SB, a composition similar to the typical clinical distribution of people with SB in Korea [30]. In other words, most of them had invisible disabilities, such as urinary and/or bowel dysfunction, but lacked any visible disability, such as gait disturbance, orthopedic deformities, or cognitive impairment [30]. Previous research on the social and emotional adjustment of Swedish adolescents with SB showed that adolescents with milder physical disabilities had the most prominent signs of social and emotional problems [31]. Adolescents without visible disabilities tend to have ambiguous identities, being at the boundary between healthy people and people with disabilities [19, 31]. For example, there may be more tension around toilet issues because their peers cannot directly observe the disability [31]. Adolescents with mild types of SB tend to evaluate themselves and compare their abilities to those of their healthy peers, which can lead to increased disappointment and frustration and require additional emotional and social adaptation [31]. In terms of the condition-severity paradox or disability paradox [32, 33], high-functioning adolescents with SB in Korea may be psychosocially vulnerable and in need of attention and support for their difficulties in transition.

Acquisition of self-management skills has been identified as a critical facilitator in the transition to independent living in adulthood. By planning and performing bladder emptying on their own, they can participate in activities such as playing with friends all day long without the need for parental supervision. This is supported by a recent study demonstrating that self-management was a precursor for the transition to adulthood for adolescents with SB [7]. Many qualitative and quantitative studies have suggested that acquiring disease-specific self-management skills and performing them independently is key to a successful transition [7, 34–37]. In addition, self-management is essential to maintaining normality in the daily life of adolescents with SB [34]. If bladder or bowel emptying is not properly managed and urinary or fecal incontinence occurs, adolescents are at risk of feeling extreme shame and psychosocial isolation and damage to their interpersonal relationships [38, 39]. These experiences can negatively affect their school life and community participation and consequently decrease their quality of life [19, 32, 34, 38, 39]. They indicate that interventions to enhance self-management skills should be provided to aid smooth transition.

There is a consensus regarding the importance of starting self-management at an early age, and parenting styles are known to play an important role in the initiation of independent self-management [13, 34, 36, 40], as shown in the current findings. However, owing to the complexity of self-management skills, such as CIC or enema, parents tend to be overprotective, and adolescents with SB tend to be highly dependent on their parents for self-management, resulting in delayed independence and transition [34]. Adolescents often experience tension between being cared for by their parents and being independent, and they worry about their future, particularly as they become adults and prepare to transition to an independent life [34]. As suggested by previous studies [6, 31, 34, 35, 37, 38], to prompt the transition of adolescents with SB, it is necessary to encourage desirable parenting styles for parents raising children with SB.

School life and peer relationships were important for adolescents with SB in this study. Consistent with previous studies, the current findings showed that peer relationships and school teachers had both positive and negative influences on their transition [31, 34, 36, 38]. These influences ranged from supportive relationships to intrusive questions about prolonged toilet use, teasing, or bullying [19, 31, 34, 36, 38]. Peer relationships are important not only for transitions to adulthood as a relational factor but also in terms of social facilitation, contributing to health-promoting behaviors [37]. In addition, as adolescents spend much time at school, efforts to improve their school environment, including raising teachers’ awareness, are needed. Previous research [17] from South Korea found performing CIC at school to be a barrier due to the lack of appropriate places and teachers’ knowledge about CIC. Adolescents with SB kept performing CIC secretly in school, with only 60% showing CIC compliance [17]. To create school environments that allow comfortable bladder management, peers’ and school teachers’ knowledge and acceptance of SB should first be established.

Participation in self-help groups can facilitate adolescents’ transition, as demonstrated in previous studies [40–42]. Such programs provided opportunities for participants in this study to experience sincere empathy through sharing common experiences with others. After participating in self-help groups, they gained insight through self-reflection and had improved social and self-management skills, facilitating their transition [43]. Additionally, mentors serve as role models for adolescents with chronic conditions or disabilities and can provide emotional and social support, motivation, and encouragement for the transition [31, 42]. Adolescents with SB should be encouraged to participate in self-help groups or mentorship programs.

According to our findings, adolescents with clinically mild SB may be more psychosocially vulnerable and experience difficulties in the transition to adulthood in the Korean cultural context. As most participants were bullied for being different, they struggled to conceal their disabilities from others. Furthermore, thoughts about being different from their friends without disabilities made participants miserable during adolescence. These experiences hindered the transition to adulthood by lowering their self-concept and hampering self-management. As culture is an important factor that can influence people’s thoughts and behaviors [16, 19, 34, 40], the sociocultural barriers identified in this study need to be addressed considering the Korean context, in which self-concept develops from an interdependent perspective [16]. Above all, self-concept, including self-esteem, self-confidence, and self-efficacy, is crucial to improve the self-management and independence of adolescents with SB [35]. Establishing a self-concept embracing one’s chronic condition or disability is also important for maintaining daily life and for growing into an independent adult [19, 34]. Therefore, it is necessary to promote self-esteem, self-efficacy, and body image while understanding Korean sociocultural characteristics (interdependent view of self-concept) that affect the transition to adulthood among adolescents with clinically mild SB.

Despite existing research, the transition experiences of adolescents with clinically mild SB and their influencing factors have not been fully explored; thus, the present findings can serve as a foundation to support their transition to adulthood. In Korea, there are few studies on the transition to adulthood among adolescents with SB, and there is no healthcare system, institutional, or policy support to develop clinical practices for their transition to adulthood [14, 15, 23]. Therefore, we suggest that, similar to Health People 2030 in the United States, supporting the transition of adolescents with chronic conditions into adulthood should be set as a public health goal in Korea and policies, healthcare systems, and practices are needed to support the same.

### Limitations

Several limitations should be acknowledged in this study. First, the findings cannot be generalized beyond the study population, that is, young adults with mild SB. As the study was conducted at a single university hospital in Korea, specific regional and institutional characteristics might have influenced the results. Second, young adults with SB who voluntarily participated in the study might have had different perspectives and experiences from those who chose not to participate. Social desirability in answers cannot be excluded. Third, quotes were translated from Korean into English; it is, therefore, possible that the meaning of translated quotes subtly differed from the original meaning in Korean. However, despite these limitations, this study offers an improved understanding of the difficulties encountered by those living with a rare and intractable condition in Korea.

## Conclusions

Korean young adults with SB shared their experiences of struggling to properly self-manage their chronic condition, particularly concerning regular bladder emptying, in their daily lives and social relationships during the transition from adolescence to adulthood. To facilitate the transition to adulthood, education on SB and self-management for adolescents and on parenting styles for their parents are important. Furthermore, to eliminate barriers to the transition to adulthood, promoting understanding of the disability among school teachers and making school restrooms CIC friendly are needed.

## Supplementary Information

**Additional file 1.** COREQ (COnsolidated criteria for REporting Qualitative research) Checklist.

## Data Availability

The data that support the findings of this study are available from the corresponding author on reasonable request.

## Abbreviations

CIC: Clean Intermittent catheterization
FGI: Focus group interview
LMMC: Lipomyelomeningocele
MMC: Myelomeningocele
SB: Spina bifida
VP shunt: Ventriculoperitoneal shunt
